# A Small Antigenic Determinant of the Chikungunya Virus E2 Protein Is Sufficient to Induce Neutralizing Antibodies which Are Partially Protective in Mice

**DOI:** 10.1371/journal.pntd.0003684

**Published:** 2015-04-23

**Authors:** Christopher Weber, Sarah M. Büchner, Barbara S. Schnierle

**Affiliations:** Department of Virology, Paul-Ehrlich-Institut, Langen, Germany; U.S. Naval Medical Research Unit No. 2, INDONESIA

## Abstract

**Background:**

The mosquito-borne Chikungunya virus (CHIKV) causes high fever and severe joint pain in humans. It is expected to spread in the future to Europe and has recently reached the USA due to globalization, climate change and vector switch. Despite this, little is known about the virus life cycle and, so far, there is no specific treatment or vaccination against Chikungunya infections. We aimed here to identify small antigenic determinants of the CHIKV E2 protein able to induce neutralizing immune responses.

**Methodology/Principal Findings:**

E2 enables attachment of the virus to target cells and a humoral immune response against E2 should protect from CHIKV infections. Seven recombinant proteins derived from E2 and consisting of linear and/or structural antigens were created, and were expressed in and purified from E. coli. BALB/c mice were vaccinated with these recombinant proteins and the mouse sera were screened for neutralizing antibodies. Whereas a linear N-terminally exposed peptide (L) and surface-exposed parts of the E2 domain A (sA) alone did not induce neutralizing antibodies, a construct containing domain B and a part of the β-ribbon (called B+) was sufficient to induce neutralizing antibodies. Furthermore, domain sA fused to B+ (sAB+) induced the highest amount of neutralizing antibodies. Therefore, the construct sAB+ was used to generate a recombinant modified vaccinia virus Ankara (MVA), MVA-CHIKV-sAB+. Mice were vaccinated with MVA-CHIKV-sAB+ and/or the recombinant protein sAB+ and were subsequently challenged with wild-type CHIKV. Whereas four vaccinations with MVA-CHIKV-sAB+ were not sufficient to protect mice from a CHIKV infection, protein vaccination with sAB+ markedly reduced the viral titers of vaccinated mice.

**Conclusions/Significance:**

The recombinant protein sAB+ contains important structural antigens for a neutralizing antibody response in mice and its formulation with appropriate adjuvants might lead to a future CHIKV vaccine.

## Author Summary

Chikungunya virus (CHIKV) is transmitted by mosquitos and causes high fever and severe joint pain in humans. It is expected to spread in the future to Europe and has recently reached the USA due to vector switch and climate change. There is no specific treatment or vaccination against CHIKV infections. However, vaccination should be an efficient way to control its spread. The CHIKV envelope glycoprotein E2 enables attachment of the virus to target cells and a humoral immune response against E2 should protect from CHIKV infections. We aimed to identify small antigens of the CHIKV E2 protein that are able to induce neutralizing antibodies. These antigens should enable the production of cost effective, safe and efficient vaccines. The surface-exposed parts of the E2 domain A (sA) fused to domain B and a part of the β-ribbon that joins domain A with B (sAB^+^) induced most effectively neutralizing antibodies and mice vaccinated with this protein were partially protected from a CHIKV challenge infection. The protein sAB^+^ was identified as a useful antigen for developing a vaccine when formulated with an appropriate adjuvant.

## Introduction

Chikungunya virus (CHIKV) is a mosquito-transmitted alphavirus that causes chikungunya fever in humans. Most CHIKV infections are symptomatic, with an incubation period of 2–4 days and the disease is characterized by sudden onset of fever, headache, malaise, arthralgias or arthritis, myalgias, and lower back pain. After the acute phase, polyarthritis can be recurrent and can persist for several years after infection [1]. This raises serious public health and economic problems during large outbreaks. So far, *Aedes aegypti* has been the most important CHIKV vector, but during a large outbreak in 2006 in La Réunion, *Aedes albopictus* (the Asian tiger mosquito) was the primary vector [2]. The more effective transmission via *Aedes albopictus* was due to only one point mutation (A226V) in the E1 envelope protein. *Aedes albopictus* also inhabits temperate and even cold temperate regions of the eastern and western hemispheres, including Europe and the United States of America. This trend will continue with escalating climate change and CHIKV will no longer be confined to developing nations [3]. There is no specific treatment for chikungunya fever and care is only supportive, based on the symptoms. No licensed CHIKV vaccine exists. Thus, there is an urgent demand for the development of a prophylactic vaccine.

Several vaccine approaches have been developed; however, so far without resulting in a market-approved vaccine. CHIKV vaccines have either been formulated as formalin-inactivated CHIKV [4] or live-attenuated CHIKV vaccine candidates like the CHIKV 181/25 strain [5]. CHIKV 181/25 is attenuated by only two point mutations and reversions in vaccinated mice have appeared, suggesting that genetic instability is the source of its reactogenicity [6]. Internal ribosome entry site (IRES)-based live-attenuated CHIKV vaccines (CHIK-IRES vaccines) should circumvent this problem and would additionally prevent vaccine spread by mosquitos [7]. Other approaches are chimeric vaccine strains based on the genetic backbones from Sindbis virus or the TC-83 vaccine strain of Venezuelan equine encephalitis virus [8,9], [9], a DNA vaccine based on codon-optimized consensus envelope protein (E1, E2 and E3) sequences [10], a VLP-based vaccine expressing the CHIKV envelope proteins [11], or recombinant measles [12]. As sterilizing protection can be reached with CHIKV-specific antibodies [13], protein-based vaccines might be envisioned. Recently, an E2 protein-based vaccine candidate has been described that is able to protect mice from CHIKV challenge infections [14]. In order to ease production of vaccine antigens, we were interested to test whether small linear antigens would be sufficient to elicit a protective immune response against CHIKV.

CHIKV is a (+) single-stranded (ss) RNA virus and enters cells by receptor-mediated endocytosis in a pH-dependent fusion step. CHIKV has two surface proteins: the two transmembrane glycoproteins E2 and E1. E1 is a class II viral fusion protein and E2 most likely mediates cell attachment, although the cellular receptor is still unknown [15]. E2 and E1 associate as trimers of heterodimers (E2–E1) on the particle surface. The ß-sheet-containing E2 protein interacts with E1, covers the hydrophobic E1 fusion loop, and forms the center of the trimer [16]. The E2 protein is subdivided into three immunoglobulin domains called A, B and C. Domains A and B are implicated in receptor binding [16], [15]. Domain B is located at the membrane distal part and forms the tip of E2. It is connected with domain A via a long ß-ribbon connector. Domain A is located at the center and domain C is close to the viral membrane and most likely not efficiently accessible to antibodies [15]. A linear epitope located at the N-terminus of E2 (aa 1–12, termed E2EP3) has been described to be the target of early neutralizing IgG responses of CHIKV-infected patients, mice and monkeys [17], [13]. This main epitope is located proximal to the furin cleavage site and is therefore prominently exposed on the surface of the virus, forming a stalk that points away from the virus envelope.

Here, we derived surface-exposed regions of E2, produced the recombinant proteins in *E*.*coli* and analyzed the induction of neutralizing antibodies. N-glycosylation of the E2 protein is not expected to interfere with antibody binding, since the two glycosylation sites in E2 are outside the regions used for vaccination. An antigen able to induce neutralizing antibodies was identified and partially protected vaccinated mice from challenge infection.

## Methods

### Cell culture

All cells used for this study were cultured at 37°C under 5% CO_2_. HEK 293T (ATCC: CRL-1573) cells were incubated in Dulbecco’s modified Eagle’s medium (DMEM; Lonza, Verviers, Belgium). BHK 21 (CCL-10) cells were grown in Roswell Park Memorial Institute medium (RPMI; Biowest, Nuaille, France). RK13 (CCL-121) cells were cultured in Eagle's Minimum Essential Medium (EMEM; Biochrom, Berlin, Germany). The used media were supplemented with 10% FBS (v/v; PAA, Pasching, Austria) and 5% L-glutamine (200 mM; Lonza, Verviers, Belgium).

### Plasmids, DNA and virus

The codon-optimized CHIKV E3-E1 gene (based on isolate “S27”) was synthesized by GeneArt (Life Technologies, Darmstadt) and cloned into the plasmid pIRES2-eGFP (Clontech/Takara, 78100 Saint-Germain-en-Laye, France) as described previously [18].

The CHIKV E2-derived constructs L and sAB^+^ were synthesized by GeneArt (Regensburg, Germany) (codon optimized, strain LR2006 derived sequences see S1 Fig) and cloned into the plasmid pET-15b (Merck Millipore [Novagen]), Darmstadt, Germany) via *Nde*I and *Bam*HI (for sAB^+^, primers see S1 Fig). Construct L contains five repeats of the sequence encoding S1- T12 linked by glycine-serine (G-S) linkers; construct sA encodes S1-T12, I56-G82, T94-H99, G114-H123 and Q158-T164 linked by G-S linkers; B^+^ encodes P172-H256. Constructs sA, B^+^, LsA, LB^+^ and LsAB^+^ where derived from the two synthesized genes via PCR and also cloned into pET-15b (already containing the L part for LB^+^, LsA and LsAB^+^) using the primers listed in the S1 Fig Sequence identities were verified by sequencing. Addition of a secretion signal to the construct sAB^+^ was implemented by cloning the synthesized gene into the vector pSecTag2 B (Invitrogen, Life Technologies, Darmstadt) via *Sfi*I and *Apa*I.

The wild-type CHIKV used for infections was a kind gift of Matthias Niedrig (Robert-Koch-Institut, Berlin, Germany) [19].

### Recombinant MVA construction

To clone the secretion signal containing construct sAB^+^ into the MVA expression plasmid pIII-pmH5, the BamHI site in the plasmid was blunted via T4-DNA polymerase. The sAB^+^ gene was excised from the pSecTag2 B vector by *Nhe*I and *Pme*I and subsequently blunted. Ligation of insert and vector resulted in the MVA vector plasmid pIII-CHIKV-sAB^+^. Recombinant MVA-CHIKV-sAB^+^ was created by parallel transfection and infection of BHK21 cells with 1 μg of plasmid DNA and MVA (wild-type) at an MOI of 0.05. Plaque selection was done on RK-13 cells as described before [20], [21]. Successful generation of the recombinant MVA-CHIKV-sAB^+^ was confirmed by PCR of MVA genomes derived from infected BHK 21 cells (S2 Fig). Virus amplification was performed on BHK21 cells as described by [22], [23].

### Protein expression und purification

Proteins were expressed in BL21-CodonPlus (DE3)-RIPL competent cells (Agilent Technologies, Böblingen, Germany) transformed with the pET-15b plasmid containing construct L, sA, B^+^, sAB^+^, LsA, LB^+^ or LsAB^+^. Bacteria were inoculated into 100 ml of LB medium containing ampicillin (0.1 mg/ml) and grown overnight (37°C, 220 rpm). After 16 hours, 2 l of LB medium were inoculated with the 100 ml overnight culture. The bacteria were grown to an OD_600_ of 0.5–0.7, then protein expression was induced by the addition of 1 mM IPTG. After another 2.5 hours of incubation, cells were harvested and the pellets were frozen at -20°C.

All recombinant proteins were purified from the bacteria pellets under native conditions using HisTrap FF Crude columns (GE Healthcare, Freiburg, Germany) and the ÄKTA system (GE Healthcare, Freiburg, Germany) as described by [24]. After purification, proteins were dialyzed against PBS using Slide-A-Lyzer Dialysis Cassettes 3.5K MWCO (Pierce; Thermo Scientific, Bonn, Germany) and concentrated with Ultra-4 3 kDa Centrifugal Filter Units (Merck Millipore, Schwalbach, Germany). The protein concentration was compared to marker proteins by 12.5 or 15.0% SDS-PAGE and adjusted to 1 mg/ml. Subsequently proteins were shock-frozen with liquid nitrogen and stored at -80°C. For experiments, proteins were thawed in a 37°C water bath. The identity of the purified proteins was confirmed by mass spectrometry (for example S3 Fig).

### Lentiviral vector particle production

Lentiviral vector particle production was performed as described previously [18]. Briefly, HEK 293T cells were seeded in 10 cm dishes in a volume of 10 ml DMEM. Cells were cotransfected 16 hours post seeding with the plasmids pCSII-Luc, pMDLg/pRRE, pRSVrev, and pHIT-G or pIRES2-eGFP-CHIKV E3–E1 using Lipofectamine 2000 (according to the manufacturer’s protocol; Life Technologies). After 24 hours incubation, the medium was discarded and replaced by 5 ml of fresh DMEM. Another 24 hours later, the supernatant containing the vector particles was harvested, sterile filtered with 0.45 μm filters (Sartorius, Göttingen, Germany), and frozen at -80°C.

### Transduction of cells with lentiviral vector particles

Cells were transduced with pseudotyped lentiviral vector particles in 384-well plates as described previously [18]. Briefly, 6000 HEK 293T cells per well were seeded (using a MultiFlo Microplate Dispenser; BioTek, Bad Friedrichshall, Germany) in 20 μl of DMEM in white CELLSTAR 384-well microtiter plates (Greiner Bio-One, Frickenhausen, Germany) and incubated for 16–24 h at 37°C. Sera were serially diluted in DMEM and vector particles (4 times; dilutions ranged from 1:30 to 1:2340) and mixed 1:1 with diluted vector particles (CHIKV Env or VSV-G pseudotyped particles in DMEM) in 96-U-well plates (Thermo Scientific, Rockford, IL, USA), and incubated at 4°C for 1 hour. This resulted in equal amounts of vector and serially diluted compound. The vector particle-sera mixtures were then added to the cells using a Matrix Multichannel Equalizer Electronic Pipette (Thermo Scientific, Rockford, IL, USA), transferring 20 μl each to three wells of the 384-well plate out of one well of the 96-well plate. This resulted in a final concentration of serum ranging from 1:60 to 1:4680 in the 384-well plate. Cells were incubated with the vector particle-sera mixtures for another 16–24 hours. Afterwards, 20 μl of BriteLite (PerkinElmer, Rodgau, Germany) substrate was added. After 5 minutes incubation at room temperature, the luciferase signal was detected using the PHERAstar FS (BMG LABTECH, Ortenberg, Germany).

### SDS-PAGE and western blot analysis

The proteins were separated by SDS-PAGE and the gel was subsequently stained with Coomassie (Bio-Rad, München, Germany). For Western blots, a Bio-Rad semi-dry blotter was used. Proteins were transferred onto PVDF membranes with a 50 mM sodium borate pH 9.0, 20% methanol, and 0.1% SDS buffer at 100 mA per membrane for 75 min. Subsequently, membranes were blocked with Roti-Block (Carl Roth, Karlsruhe, Germany). Proteins were detected with an anti-myc antibody (BD Pharmingen, Heidelberg, Germany), or with a ß-actin antibody (Sigma, Munich, Germany). Detection was performed with the ECL system (Amersham, Freiburg).

### CHIKV RNA isolation and RT PCR

Viral RNA was isolated from mouse sera and organs with the QIAamp Viral RNA Mini Kit and the RNeasy Lipid Tissue Mini Kit (Qiagen, Hilden, Germany), respectively, according to the manufacturer’s protocol. CHIKV RNA levels were tested with the RealStar Chikungunya RT-PCR Kit 1.0 (Altona Diagnostics, Hamburg, Germany). The kit was used according to the manufacturer’s protocol. The readout was performed using a LightCycler 480 Instrument II (Roche, Basel, Switzerland).

### Mouse experiments

Mice (female, BALB/c) (Janvier, Saint-Berthevin Cedex, France) were kept at the Paul-Ehrlich-Institut in ventilated cages. They were first immunized at an age of seven weeks. Each mouse was injected subcutaneously in the neck region with 100 μg of protein in PBS mixed 1:1 with alum (Alhydrogel 2%; InvivoGen, San Diego, California, USA) per immunization. Blood was collected immediately before the immunizations and after the final immunization. Infection was carried out intranasally with 1 × 10^6^ PFU CHIKV in 30 μl PBS. Blood was collected on day two and four post infection and mice were sacrificed on day four post infection and organs were collected. Sera were obtained from blood using Microtainer SST tubes (BD, San Diego, CA, USA). For RNA isolation, sera were directly frozen at –80°C. Organs were also frozen at -80°C in 1 ml RNA*later* RNA Stabilization Reagent (Qiagen, Hilden, Germany), according to the manufacturer’s protocol. For the luciferase neutralization assay, sera were first incubated at 56°C for 30 minutes and then frozen at -80°C. All experiments were performed in accordance with German legal requirements.

### Ethics statement

Experiments were performed in accordance to legal requirements (German protection of animals act (deutsches Tierschutzgesetz) and experimental animal regulation (Tierschutz-Versuchstierverordnung)) and after approval of the regional council Darmstadt, Germany (permit number V54 19c 20/15 F107/123).

### Statistical analysis

Statistical analyses were done using the GraphPad Prism 5.04 software (La Jolla, CA, USA). For the p-values of the neutralization assay, the paired two-tailed t-test was performed. For the viral titers’ p-values and ELISA analysis of anti-CHIKV antibody reactivities, the unpaired two-tailed t-test was performed.

## Results

### Derivation of E2 subunits containing surface-exposed domains

**Fig 1:**
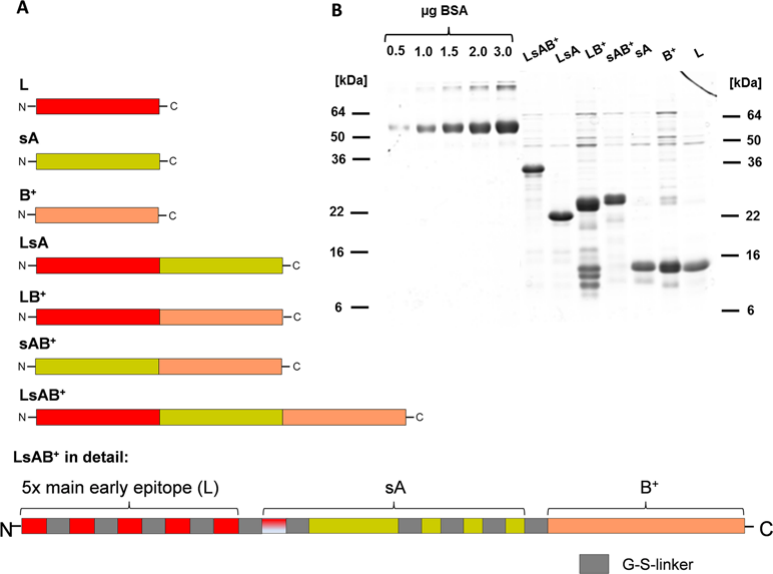
Summary of the recombinant genes and the proteins purified from *E*.*coli*. A: Schematic representation of the combinations of E2 fragments. Red- main early neutralizing epitope; yellow- surface exposed domains of A; red/white—one peptide of L; orange- domain B with ß-ribbon connector. B: Overview of the recombinant proteins purified by Ni^2+^ affinity chromatography from *E*.*coli*. Proteins were separated on a 15% SDS-PAGE and stained with Coomassie. The prominent faster migrating bands of protein LB^+^ were identified by mass spectrometry as degradation products of LB^+^ (see S3 Fig). Lanes 1–5 from the left were loaded with the indicated amounts of BSA and used to estimate the protein amount of the recombinant proteins.

The CHIKV E2 protein is responsible for binding to the still unidentified cellular receptor. It is organized in three domains named A, B and C, where C is unlikely to be able to interact with antibodies [25] and was therefore excluded from the analysis. Based on the crystal structure of the E2 protein [15], putatively surface-exposed, linear antigens of domain A were chosen (including one 12 amino acid subunit of the L construct) and artificially assembled and linked by G-S linkers. This resulted in a small protein named sA (surface-exposed A). The entire domain B including a part of the ß-ribbon connector implied to be surface exposed was used unaltered to produce construct B^+^. In addition, the linear epitope located at the N-terminus of E2 (aa 1–12), described to be the main target of early neutralizing IgG responses of CHIKV infected patients [17], was used to construct a recombinant gene containing a multimer of five of these peptide sequences joined by G-S linkers. This construct was named L. The sequences of these constructs are provided in the Supplements. Additionally, the three components were combined with each other as illustrated in Fig 1A, resulting in seven recombinant genes containing a 6-histidine tag originating from the expression vector for protein purification. The resulting proteins were expressed in *E*.*coli* and purified under native conditions by Ni^2+^-affinity chromatography. Fig 1B gives an overview of the purified proteins separated by SDS-PAGE and stained with Coomassie.

### Vaccination of BALB/c mice

**Fig 2:**
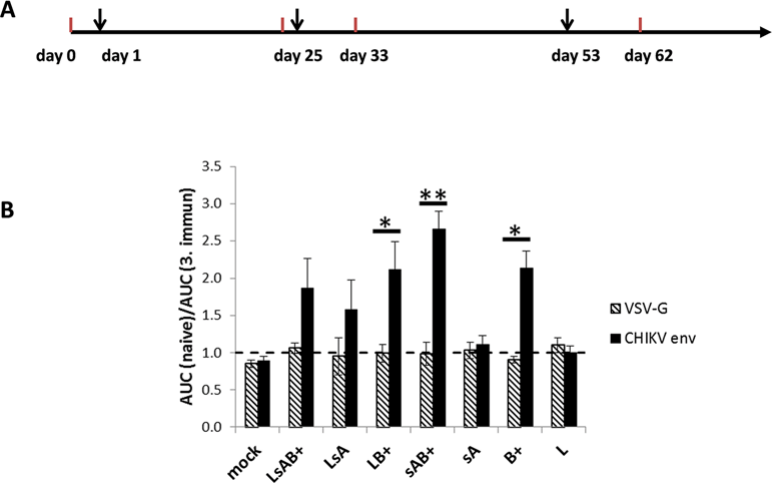
CHIKV neutralizing activity of mouse sera. A: Schematic representation of the immunization schedule. Immunizations are indicated by an arrow and blood sampling by a red line. B: Neutralization assay with mouse sera. Serum of immunized mice was serially diluted, incubated with CHIKV Env or VSV-G pseudotyped vector particles and analyzed for its neutralizing activity by detection of relative luciferase activities. The AUC of the luciferase activity obtained with preimmune serum was divided by the values obtained with serum after the last immunization. The data represent the mean values of three mice and * and ** indicate statistical significance. Statistical analyses were done using the GraphPad Prism 5.04 software and the p-values were determined by the paired two-tailed t-test.

The recombinant proteins were subsequently tested for their ability to induce neutralizing antibodies (nAb). Female BALB/c mice (n = 3 per recombinant protein) were immunized at an age of seven weeks. Additional immunizations were performed on days 25 and 53 after the first immunization. For each immunization, 100 μg of recombinantly expressed protein in PBS were mixed 1:1 with alum and injected subcutaneously into the neck region of the mice. Blood was always collected before each immunization and nine days after the last immunization (Fig 2A). First, the sera obtained after the third immunization were evaluated by ELISA to confirm seroconversion. Except one mouse vaccinated with LsA, all mice developed IgG antibodies against the immunogen, although to varying extends (S4 Fig). The proteins LsA, sA and L developed only weak CHIKV reactivity, however LsAB^+^, LB^+^ sAB^+^ and B^+^ were highly CHIKV-positive. Subsequently, the sera were analyzed for the presence of neutralizing antibodies by a neutralization assay based on blocking the entry of CHIKV-Env-pseudotyped lentiviral vectors encoding luciferase [18]. Transduction of HEK 293T cells with VSV-G-pseudotyped vectors served as a control for CHIKV specificity. The mouse sera were diluted 1:60 to 1:4680 and the area under the curve (AUC) of luciferase units obtained with preimmune sera was divided by the AUC obtained with sera after the third immunization (Fig 2B). Three mice were analyzed per construct and the mean values are given. As expected, transduction by VSV-G-pseudotyped vector particles was not affected by the mouse sera, demonstrated by an AUC ratio of 1 (Fig 2B). Likewise the recombinant proteins sA and L did not induce nAb. However, a statistically significant induction of neutralizing antibodies compared to VSV-G-pseudotyped vectors was detected in sera of LB^+^-, sAB^+^- and B^+^-immunized mice. The protein sAB^+^ induced the highest amount of nAb, illustrated by an AUC ration of 2.7. The sA protein apparently enhanced the induction of neutralizing antibodies in combination with domain B^+^, although sA alone did not induce nAb. Altogether these data show that B^+^ was necessary and sufficient to induce nAbs in mice.

### Generation of a recombinant MVA expressing the protein sAB^+^

**Fig 3:**
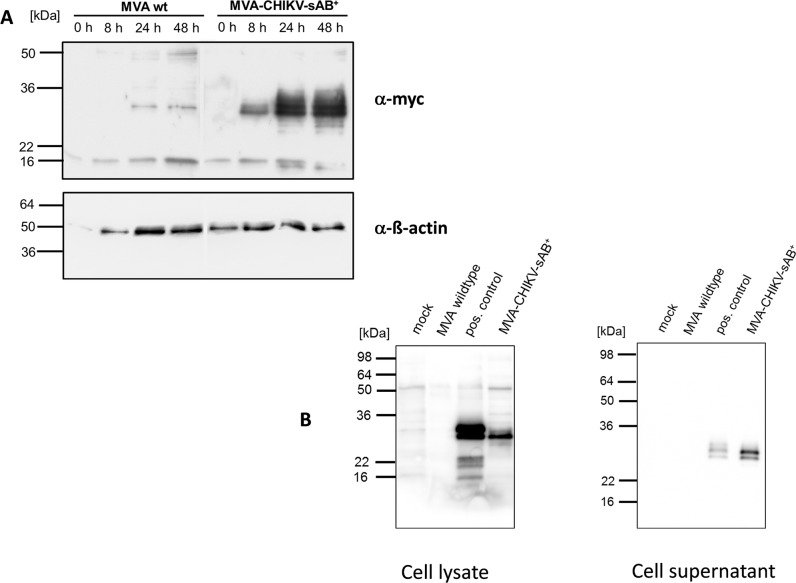
Characterization of MVA-CHIKV-sAB^+^. A: Expression of the recombinant sAB^+^ protein. HEK 293T cells were infected with MVA wt or MVA-CHIKV-sAB^+^ at a MOI of 5 and the cells were harvested at the indicated time points. The cell lysates were analyzed by Western blot with an antibody directed against the myc-tag. Equal loading was controlled by detection of ß-actin (lower panel). B: sAB^+^ is secreted from cells. HEK 293T cells were infected at a MOI of 0.1 and harvested after 72 hours. Cell lysates and supernatants were analyzed by Western blot with an antibody directed against the myc-tag. Transiently transfected/MVA infected cells served as a positive control.

To further evaluate the protein sAB^+^ as a potential CHIKV vaccine, we attempted to increase the immune responses directed against sAB^+^ by generating a recombinant vaccine virus based on the highly attenuated strain modified vaccinia virus Ankara (MVA). MVA allows high foreign protein expression although the virus does not replicate in human cells, giving it a very good safety profile. A eukaryotic secretion signal and a myc-tag was added to the gene encoding sAB^+^ and the construct was subsequently cloned into the MVA expression vector pIII-mH5 [26] generating the plasmid pIII-CHIKV-sAB^+^, where expression is controlled by a strong early/late promoter. The recombinant virus, MVA-CHIKV-sAB^+^, was constructed as described previously, using K1L selection [20], [26]. Quality control was performed by PCR. Wild-type MVA and MVA-CHIKV-sAB^+^ had the same growth kinetics in permissive cells (S5 Fig). Western blot analysis confirmed the proper expression of the sAB^+^ protein to high levels at late time points in cells infected with MVA-CHIKV-sAB^+^ (Fig 3A). To demonstrate that the sAB^+^ protein is secreted, we additionally analyzed cell lysates and supernatants of MVA-infected cells 72 hours after infection. Fig 3B shows a clear band in the MVA-CHIKV-sAB^+^ samples at the expected size in cell lysates as well as in supernatants, which indicates that the protein is secreted by infected cells.

### Protective efficacy of subunit vaccines compared to recombinant MVA

**Fig 4:**
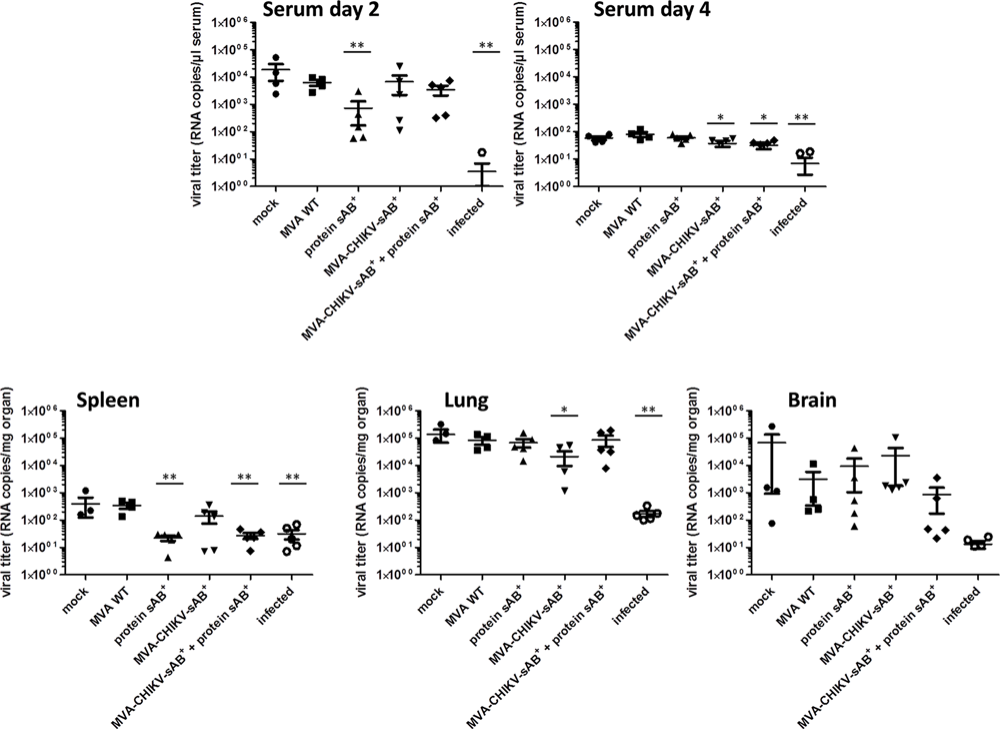
Challenge infection of immunized mice. Mice (n = 5 per CHIKV antigen immunized group, n = 4 for the control groups) were immunized with the indicated antigens and infected two weeks after the final immunization with 1x10^6^ IU CHIKV. Two and four days after infection, the CHIKV titer was determined as RNA copies/μl serum by RT-PCR. At the end of the experiments, spleen, lung and brain were isolated and the CHIKV titer was determined as RNA copies/mg organ by RT-PCR. Lane 6 shows data from mice that were infected seven weeks before and also subjected to a challenge infection (n = 5). * indicates statistical significance. Statistical analyses were done using the GraphPad Prism 5.04 software and the p-values were determined by the unpaired two-tailed t-test was performed. * P ≤ 0.05; ** P ≤ 0.01. Indicated on the x-axis are the recombinant proteins or vaccinia viruses used for immunization.

CHIKV challenge infections were again implemented in BALB/c mice. Although in this model the animals show no clinical symptoms, temporal CHIKV replication can be observed in target organs (muscle, brain, spleen) and in blood. It has been shown before that the intranasal CHIKV infection resulted in viremia lasting 2–3 days, while intraperitoneal infection yielded less consistent results [27], [28]. We tested whether a protective immune response could be induced with the sAB^+^ protein, formulated as a protein vaccine, a recombinant MVA vaccine candidate or a mixture of both. Again, female BALB/c mice (n = 5 for the CHIKV antigen groups, n = 4 for the control groups) were immunized at an age of seven weeks. Additional immunizations were then performed three, four, and six weeks after the first immunization. Each mouse was injected subcutaneously in the neck region with either 100 μg of protein in PBS mixed 1:1 with alum as before or 1 x 10^8^ PFU MVA-CHIKV-sAB^+^ per immunization. The mixed immunization was performed as a MVA-CHIKV-sAB^+^ (1 x 10^8^ PFU) prime, followed by three immunizations of a cocktail of protein/alum/MVA-CHIKV-sAB^+^ as boosts. All mice immunized with MVA-CHIKV-sAB^+^ and/or sAB^+^ protein seroconverted and were CHIKV-Env-positive when tested by ELISA with recombinant protein B^+^ as an immunogen (S6 Fig). The CHIKV-directed reactivity was higher in sAB^+^ vaccinated animals compared to MVA-CHIKV-sAB^+^ vaccinated animals. Although statistically significance was only detected for the combined MVA-CHIKV-sAB^+^/sAB^+^ protein vaccinated mice (S6 Fig). Two weeks after the final immunization, the mice were infected intranasally with 1x10^6^ PFU CHIKV in 30 μl PBS. Blood was collected two and four days post infection and the CHIKV titer in the sera, lung, spleen and brain was determined by RT-PCR. Fig 4 shows the viral titers as RNA copies in serum. A statistically significant reduction of viral titers compared to animals vaccinated with MVA wt was only detected after two days of infection in the sera of mice vaccinated with the sAB^+^ protein alone (Fig 4, lane 3). Mice that had been infected with CHIKV seven weeks before and challenged with a CHIKV infection, however, were fully protected (Fig 4, lane 6), indicating that protein sAB^+^ vaccination was not fully protective in this model. MVA-CHIKV-sAB^+^-vaccinated animals did not show any significant alteration in viral titers compared to control animals (mock or MVA wt) and the combination of MVA-CHIKV-sAB^+^ with protein did not significantly enhance the immune responses of MVA-CHIKV-sAB^+^. However, single mice in every vaccinated group exposed to CHIKV antigen showed reduced CHIKV titers, indicating some protective activity.

The intranasal infection causes in a short time virus replication in the animals, which resulted in very low viral titers in serum four days after infection in all groups. However there was a statistically significant decrease in viral titer in MVA-CHIKV-sAB and MVA-CHIKV-sAB/protein sAB vaccinated animals although the difference between the mean values was rather low. Analysis of organs at four days after infection revealed a statistically significant decrease in viral titers in spleens of mice vaccinated either with sAB^+^ protein or MVA-CHIKV-sAB^+^/protein sAB^+^ and in preinfected mice (Fig 4). In lung and brain only the preinfected mice showed a drastic decrease in viral titers (Fig 4). In lungs of MVA-CHIKV-sAB^+^ vaccinated mice a statistically significant decrease in CHIKV titer compared to MVA vaccinated mice could be observed.

## Discussion

Vaccination is considered to be the most efficient way to control the spread of CHIKV. Effective experimental vaccines have been reported before, such as attenuated CHIKV strains, inactivated CHIKV or virus-like particles (VLP). These approaches are problematic because of vaccine safety or require either large scale production of CHIKV in a BSL-3 laboratory or cell-based VLP production, both economically inconvenient processes. Adoptive transfer studies indicate that neutralization of viruses by anti-CHIKV antibodies protects against CHIKV infection [29], [13]. Therefore subunit protein vaccines might constitute safe, easy to produce, and economically favorable vaccines against CHIKV. Subunit vaccines are based only on a portion of the virus and elicit humoral immune responses. Approaches using the CHIKV E1/E2 proteins expressed in *E*.*coli* have shown promising protection of mice from CHIKV infections [30], [14]. Kumar et al. showed that recombinant E2 protein adjuvanted with alum protected mice from a challenge infection to a similar extend as inactivated CHIKV [14]. Also recombinantly expressed E1 as well as E2 proteins elicited a protective immune response [30].

Here we attempted to minimize the antigens of E2 and identify concise antigens able to induce neutralizing antibodies and confer protection. The antigens were designed by taking into account the observation that mutations in the alphavirus E2 domain A and B confer escape from neutralizing antibodies, affect tissue tropism and host range [15]. In a previous study [17], mice were vaccinated with the early, immunodominant N-terminal E2EP3 peptide coupled to KLH which induced a mild neutralizing activity and partial protective responses *in vivo*. Based on these data, we designed a pentamer of the E2EP3 peptide linked by G-S-linkers (L). Unfortunately this polypeptide did not induce any neutralizing antibodies upon vaccination of mice, although anti-L antibodies were detectable by ELISA. The E2PE3 epitope is located proximal to the E2–E3 furin cleavage site and is therefore prominently exposed on the surface of the virus to make the side accessible to the furin protease, suggesting that it is also susceptible to antibody binding. However, in contrast to other data [17], we did not observe the induction of neutralizing antibodies, which might be attributable to differences in antigen delivery or the adjuvants used for vaccination.

The second attempt to generate a synthetic polypeptide with surface exposed antigens of the E2 domain A (sA) also failed. Although the vaccination induced sA-specific antibodies, as before with the L polypeptide, no CHIKV neutralizing activity was detected in sera of immunized mice. In spite of this, the E2 domain B^+^ and combinations thereof with L or sA produced CHIKV neutralizing activity. The addition of sA may have stabilized the protein and resulted in higher neutralizing activities; nevertheless the main antigenic determinant must be located in domain B^+^. Domain B^+^ contains domain B, which covers the fusion loop in the E2–E1 dimer and prevents premature membrane fusion, and also the C-terminal part of the β-ribbon connector to domain A, a part of the acid-sensitive region (ASR). During the low pH fusion step, domain B and the ASR become disordered [16]. Antibodies binding to the ASR region or domain B could prevent the dissociation of E2 from E1 following pH triggering, reducing fusion efficiency and CHIKV entry. Domain B might also be involved in viral attachment to the cell surface [16]. Therefore, antibodies directed against domain B could also prevent the binding of viral particles to the cell. Thus, the superior potential of construct B^+^ to induce neutralizing antibodies might be due to its ability to induce antibodies which prevent viral attachment and fusion at the same time.

In a first attempt to show proof of principle that sAB^+^ is a suitable antigen for vaccine development, we generated a recombinant MVA expressing sAB^+^ as a secreted protein to enhance antigen delivery and perhaps boost the induction of cellular and humoral immunity at the same time. MVA is a highly attenuated vaccinia virus strain with a high safety profile suitable for clinical application in immunosuppressed patients [31], [32]. MVA does not replicate in human cells but shows high protein expression and, consequently, has a very good safety profile without compromising vaccination efficiency. MVA vaccination generates antigen specific cellular and humoral immunity [23]. The protein vaccine candidate was compared to MVA-CHIKV-sAB^+^ and combinations of MVA-CHIKV-sAB^+^ and protein sAB^+^. Adult mouse models for CHIKV infections are still not well established and immune responses can only be analyzed in mice that do not develop disease [33]. We chose intranasal challenge for infection of BALB/c mice which allows temporal CHIKV replication to be observed in target organs and in blood [14]. Other groups use A129 mice that are deficient in α/ß interferon signaling or C57BL/6 mice and different application routes like i.m., i.p. or i.v., which makes it difficult to compare vaccine efficacy from published data. Here, in general, CHIKV-specific vaccinated mice showed a trend to lower CHIKV replication in serum at day two after infection compared to mock or MVA wt vaccinated animals. However, a significant reduction (25-fold) in mean CHIKV titers was only observed in protein sAB^+^ vaccinated animals. Preceding infection of mice with CHIKV conferred a sterilizing immunization to the animals. In spleen, a significant reduction of viral titers was observed in protein sAB^+^ vaccinated animals as well as in MVA-CHIKV-sAB^+^ and protein sAB^+^ vaccinated animals. Furthermore, MVA-CHIKV-sAB^+^ vaccinated animals had a slightly lower viral titer in the lungs. In summary, this indicates that MVA-CHIKV-sAB^+^ vaccination was also immunogenic however with much lower efficiency than protein sAB^+^. The induction of humoral immune responses seems to be more efficient with protein vaccination than with MVA—derived delivery. Vaccination with recombinant E1 and E2 protein also only lowered the viral titer in blood after challenge infections in another study, although brain and muscle were completely protected [30]. Recently, several recombinant MVA expressing CHIKV antigens have been described [34], [35], [36] and all shown protection of vaccinated mice from a CHIKV challenge infection. MVA-CHIKV for instance expresses the CHIKV proteins C, E3, E2, 6K and E1 [35]. E2 was membrane anchored but formed no VLPs with the capsid C. MVA-CHIKV was shown to be highly immunogenic and triggered CHIKV-specific CD8^+^ T cell responses and high titers of neutralizing antibodies against CHIKV. A single dose of MVA-CHIKV protected all mice from challenge with CHIKV [35]. The differences to our data might arise either from the different antigen, the experimental setting or the MVA backbone used. MVA-CHIKV is based on a MVA variant that has immunomodulatory genes (*C6L*, *K7R* and *A46R*) deleted. MVA-CHIKV-sAB^+^ produces secreted proteins and was applied subcutaneously, MVA-CHIKV was applied intraperitoneally at a 10-fold lower dose. Challenge infections were done either intranasally or subcutaneously in the dorsal side of each hind foot. CHIKV titers were analyzed by plaque assays and, in our experiments, by the more sensitive RT-PCR. The role of cytotoxic T cells in CHIKV infections is still not well understood; however, these data imply that cytotoxic T cells induced by MVA-CHIKV may contribute to viral clearance. Only the side by side testing of all recombinant MVA expressing CHIKV antigens with the same mouse model will give clear indications on the efficacy of the vaccines.

In summary, we showed that the use of the small linear epitope L or surface exposed parts of A are not sufficient to induce a protective immune response, even if the L epitopes have been shown to be a main early target of antibodies in infected individuals. In contrast, linear antigens of domain A fused to the construct B^+^ (sAB^+^) were sufficient to induce neutralizing antibodies and to partly protect immunized mice from viremia. Most likely, B^+^ alone plays a much more prominent role in this than sA. Considering the high CHIKV dose used for the challenge infection, it might be expected that even in the formulation as protein and alum, the vaccination might be sufficient to protect against a mosquito derived CHIKV infection. However, optimization of antigen delivery might be useful and this small polypeptide might be an appropriate antigen for the development of a CHIKV vaccine.

## Supporting Information

S1 Fig Sequences and primer sequences used to generate the recombinant E2 constructs. Sequences of the constructs L and sAB^+^ were synthesized by GeneArt (Regensburg, Germany). Amino acid (aa) sequences are derived from CHIKV strain LR2006). L contains five repeats of aa S1- T12; linked by G-S linkers (G_4_SG_4_). sA: S1–T12, I56-G82, T94-H99, G114-H12, Q158-T164: linked by G-S linkers and B^+^ (P172-H256). Using the given primers the other constructs were generated by polymerase chain reactions and the above mentioned constructs as template. Fw = forward primer, rev = reverse primer.
(TIF)

S2 Fig Validation of the correct genetic organization of MVA-CHIKV-sAB^+^. BHK-21 cells were seeded in 6-well plates, and infected with MVA-CHIKV-sAB^+^ (MOI 0.1). 72 hours later, the genomic DNA of the cells was isolated. It was used as a template in a PCR with the appropriate primers for del III and *C7L* (A), and for *K1L* and the *sAB* ^*+*^ transgene (amplified by using the primers del lII for and ChW35) (B). *K1L* was only present in the plasmid control. The weak bands in the other samples are most likely due to DNA contamination, as the negative controls also show the same weak signal. H_2_O, the DNA of mock- and MVA wt-infected cells, respectively, and the plasmid DNA pIII-CHIKV-sAB^+^ were used as controls. The PCR products were loaded onto an agarose gel, the DNA was stained, and the detection was carried out using UV light. Primers used for the characterization of recombinant MVA C7L for: ATGGGTATACAGCACGAATTC; C7L rev: CATGGACTCATAATCTCTATAC; Del III for: GTACCGGCATCTCTAGCAGT; Del III rev TGACGAGCTTCCGAGTTCC; K1L int-1: TGATGACAAGGGAAACACCGC; K1L int-2 GTCGACGTCATATAGTCGAGC; ChW35 (transgene *sAB* ^*+*^) int rev: TGGCCTCCTCCTCCGCTG; M_AB fw: AAAAGTCGACAGCACCAAGGACAACTTCAAC; M_AB rev: AAAAGGATCCTCAGTGGATCTTGCCCTTCCGG
(TIF)

S3 Fig Confirmation of protein identity by mass spectrometry. Protein bands as indicated were excised from silver-stained gels and treated as described before [20]. Data analysis was performed with the Protein Lynx Global Server Version 2.3 (Waters). Peptide hits in the recombinant proteins are indicated in bold letters. The data confirm that the lower bands (LB^+^.1/2/3) are degradation product of the full length protein (LB^+^.0).
(TIF)

S4 Fig Analysis of CHIKV-specific antibodies in mouse sera tested by ELISA. The 96-well microtiter plates were coated with 60 μl CHIKV-AG (Anti-CHIKV virus IgG Elisa kit, Abcam (#ab177835); Cambridge, UK) per well by overnight incubation at 4°C. The plates were washed twice with washing buffer (PBS, 0.05% Tween20) and blocked with blocking buffer (PBS, 1% BSA) for 4 hrs. Afterwards, mouse serum at different dilutions in blocking buffer was added to the wells and the plates were incubated for 1 h at 37°C. Serum of untreated mice was used as negative control. After four times washing, horseradish peroxidase-conjugated goat anti-mouse IgG antibody (Dianova, Hamburg, Germany; 1:5.000) was added and the plates were incubated for 1 h at 37°C. After washing (4 times), 100 μl TMB solution (Tetramethylbenzidine, Interchem, Pfaffen-Schwabenheim, Germany) was used to detect the color development. After incubation for 10 min, the reaction was stopped by adding 100 μl 2N sulfuric acid. The color intensity was measured at 450 nm in a Tecan Genion Plus ELISA Plate Reader and is indicated as relative OD. The immunogens used to vaccinate the mice are indicated on the x-axis. Sera of single mice were used for the ELISA assay and their values are indicated as dots. The bar indicates the mean values of 3 mice vaccinated and the standard deviations are given as vertical bars. P-values calculated by an unpaired-students T-test are indicated as * (P ≤ 0.05) and ** (P ≤ 0.01) and show significance. A: sera were diluted 1:50. B: sera were diluted 1:100.
(TIF)

S5 Fig Growth analysis of MVA-CHIKV-sAB^+^ in primate and non-primate cell lines. The permissive cell lines DF-1 and BHK-21, and the non-permissive HeLa cells were seeded in 6-well plates and infected with MVA wt (A), or MVA-CHIKV-sAB^+^ (B) with an MOI of 0.5. Viral titers were determined at the indicated time points.
(TIF)

S6 Fig Analysis of CHIKV-specific antibodies in mouse sera tested by ELISA. The 96-well microtiter plates were coated with 100 ng protein B^+^ per well by overnight incubation at 4°C. The plates were washed twice with washing buffer (PBS, 0.05% Tween20) and blocked with blocking buffer (PBS, 1% BSA) for 4 hrs. Afterwards, mouse serum at different dilutions in blocking buffer was added to the wells and the plates were incubated for 1 h at 37°C. Serum of untreated mice was used as negative control. After four times washing, horseradish peroxidase-conjugated goat anti-mouse IgG antibody (Dianova, Hamburg, Germany; 1:5.000) was added and the plates were incubated for 1 h at 37°C. After washing (4 times), 100 μl TMB solution (Tetramethylbenzidine, Interchem, Pfaffen-Schwabenheim, Germany) was used to detect the color development. After incubation for 10 min, the reaction was stopped by adding 100 μl 2N sulfuric acid. The color intensity was measured at 450 nm in a Tecan Genion Plus ELISA Plate Reader and is indicated as relative OD. The immunogens used to vaccinate the mice are indicated on the x-axis. Sera of single mice were used for the ELISA assay and their values are indicated as dots. The bar indicates the mean values of 5 mice (4 for control groups) vaccinated and the standard deviations are given as vertical bars. P-values calculated by an unpaired-students T-test are indicated as * (P ≤ 0.05) and show significance. A: sera were diluted 1:100. B: sera were diluted 1:200. Statistically significance compared to MVA wt vaccinated mice, was only detected for MVA-CHIKV-sAB^+^/protein sAB^+^ vaccinated mice.
(TIF)
