# Supplementary figures and images for: A Small Antigenic Determinant of the Chikungunya Virus E2 Protein Is Sufficient to Induce Neutralizing Antibodies which Are Partially Protective in Mice
Source: PLoS Negl Trop Dis. 2015 Apr 23;9(4):e0003684. doi: 10.1371/journal.pntd.0003684 (PMC4407984; doi:10.1371/journal.pntd.0003684)

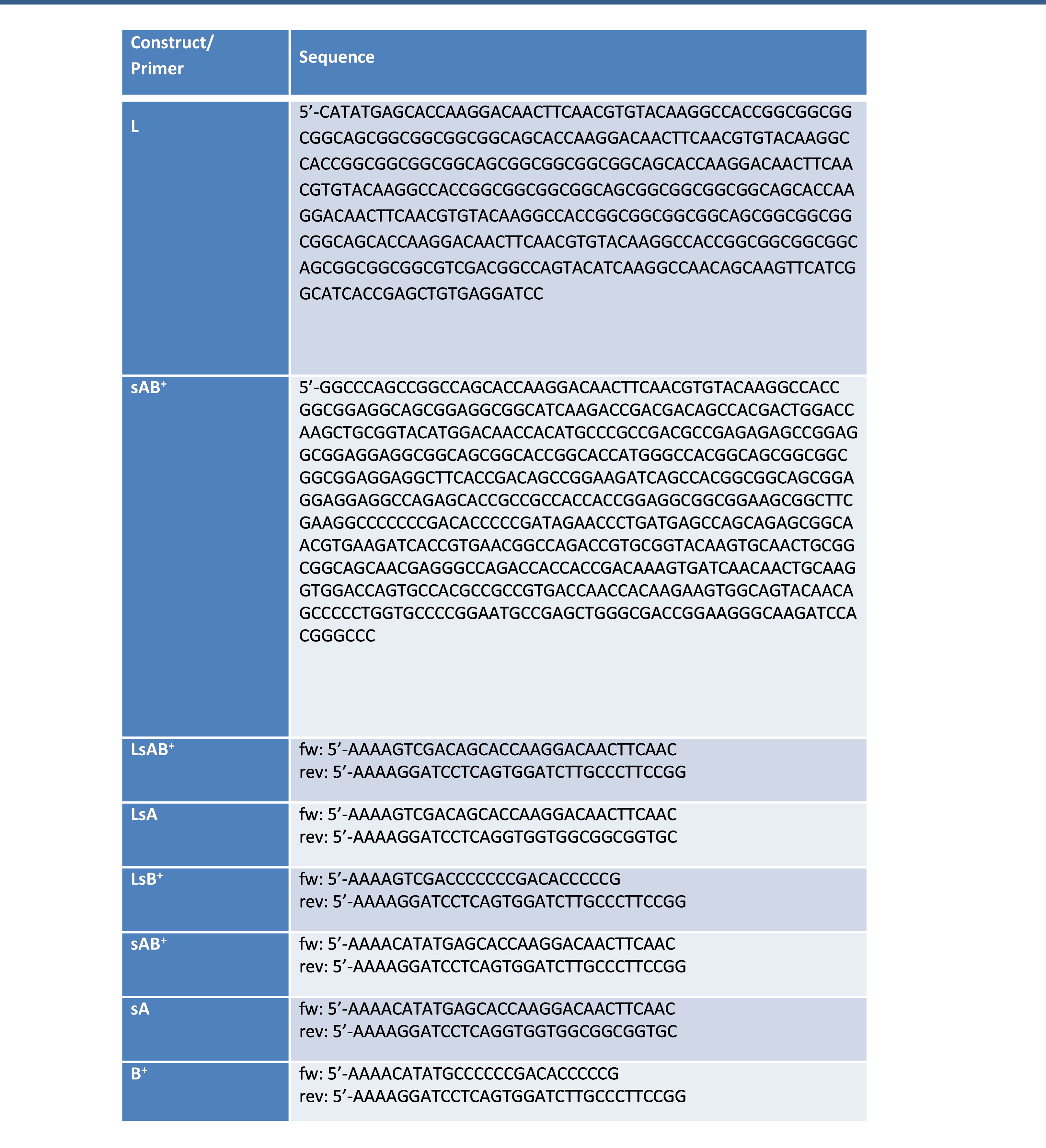

Supplement: S1 Fig — Sequences of the constructs L and sAB+ were synthesized by GeneArt (Regensburg, Germany). Amino acid (aa) sequences are derived from CHIKV strain LR2006). L contains five repeats of aa S1- T12; linked by G-S linkers (G4SG4). sA: S1–T12, I56-G82, T94-H99, G114-H12, Q158-T164: linked by G-S linkers and B+ (P172-H256). Using the given primers the other constructs were generated by polymerase chain reactions and the above mentioned constructs as template. Fw = forward primer, rev = reverse primer. (TIF) [file pntd.0003684.s001.tif]

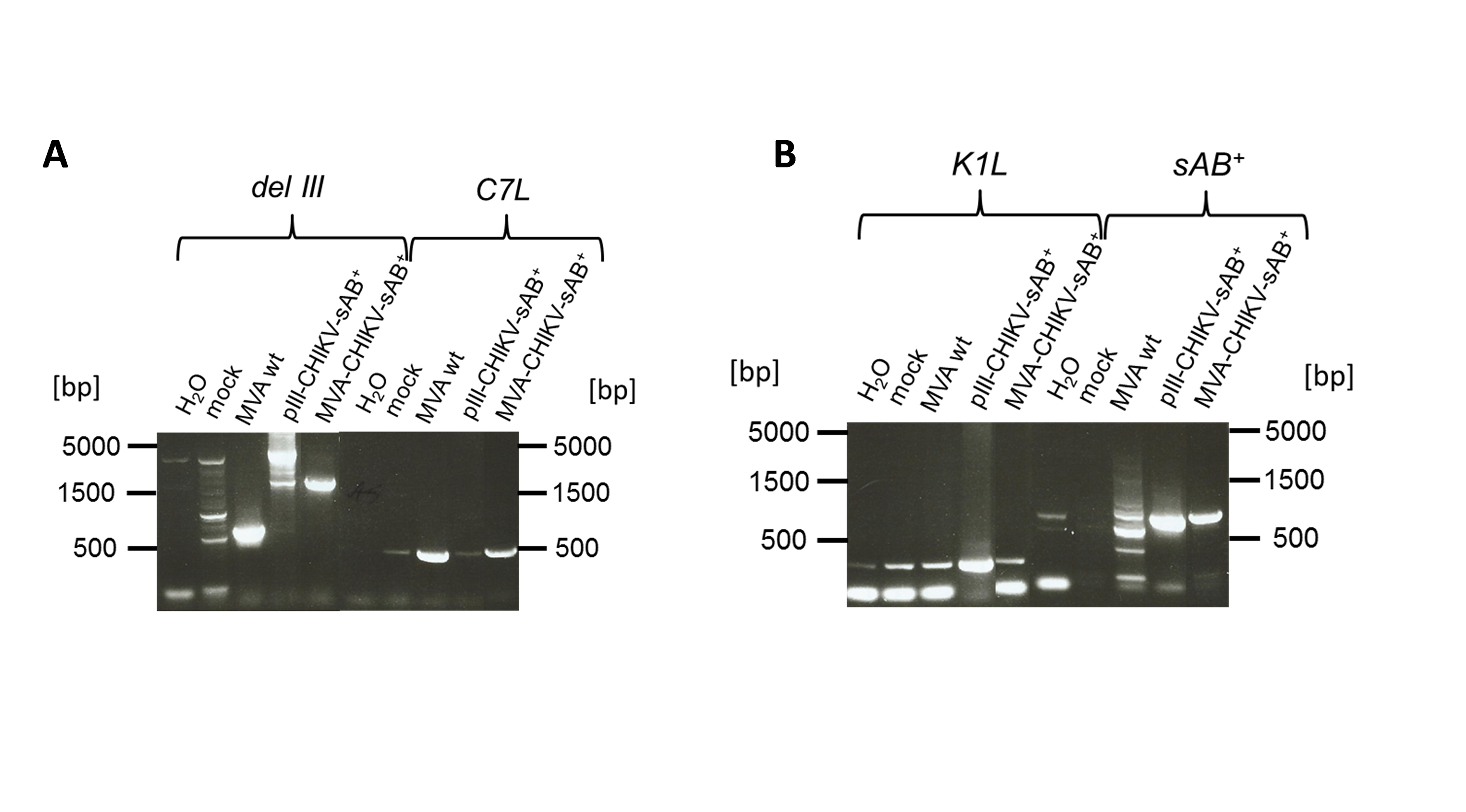

Supplement: S2 Fig — BHK-21 cells were seeded in 6-well plates, and infected with MVA-CHIKV-sAB+ (MOI 0.1). 72 hours later, the genomic DNA of the cells was isolated. It was used as a template in a PCR with the appropriate primers for del III and C7L (A), and for K1L and the sAB + transgene (amplified by using the primers del lII for and ChW35) (B). K1L was only present in the plasmid control. The weak bands in the other samples are most likely due to DNA contamination, as the negative controls also show the same weak signal. H2O, the DNA of mock- and MVA wt-infected cells, respectively, and the plasmid DNA pIII-CHIKV-sAB+ were used as controls. The PCR products were loaded onto an agarose gel, the DNA was stained, and the detection was carried out using UV light. Primers used for the characterization of recombinant MVA C7L for: ATGGGTATACAGCACGAATTC; C7L rev: CATGGACTCATAATCTCTATAC; Del III for: GTACCGGCATCTCTAGCAGT; Del III rev TGACGAGCTTCCGAGTTCC; K1L int-1: TGATGACAAGGGAAACACCGC; K1L int-2 GTCGACGTCATATAGTCGAGC; ChW35 (transgene sAB +) int rev: TGGCCTCCTCCTCCGCTG; M_AB fw: AAAAGTCGACAGCACCAAGGACAACTTCAAC; M_AB rev: AAAAGGATCCTCAGTGGATCTTGCCCTTCCGG (TIF) [file pntd.0003684.s002.tif]

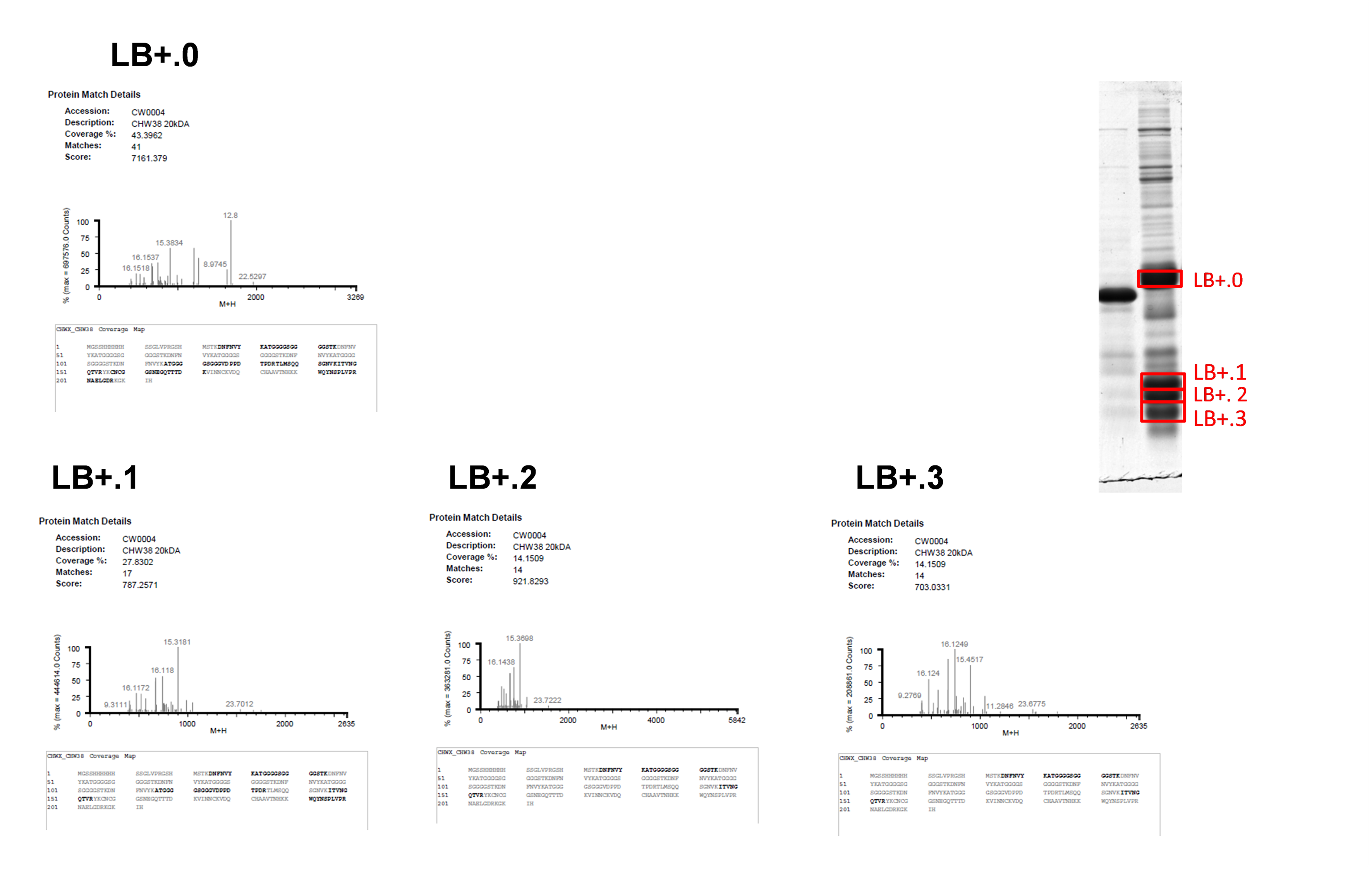

Supplement: S3 Fig — Protein bands as indicated were excised from silver-stained gels and treated as described before [20]. Data analysis was performed with the Protein Lynx Global Server Version 2.3 (Waters). Peptide hits in the recombinant proteins are indicated in bold letters. The data confirm that the lower bands (LB+.1/2/3) are degradation product of the full length protein (LB+.0). (TIF) [file pntd.0003684.s003.tif]

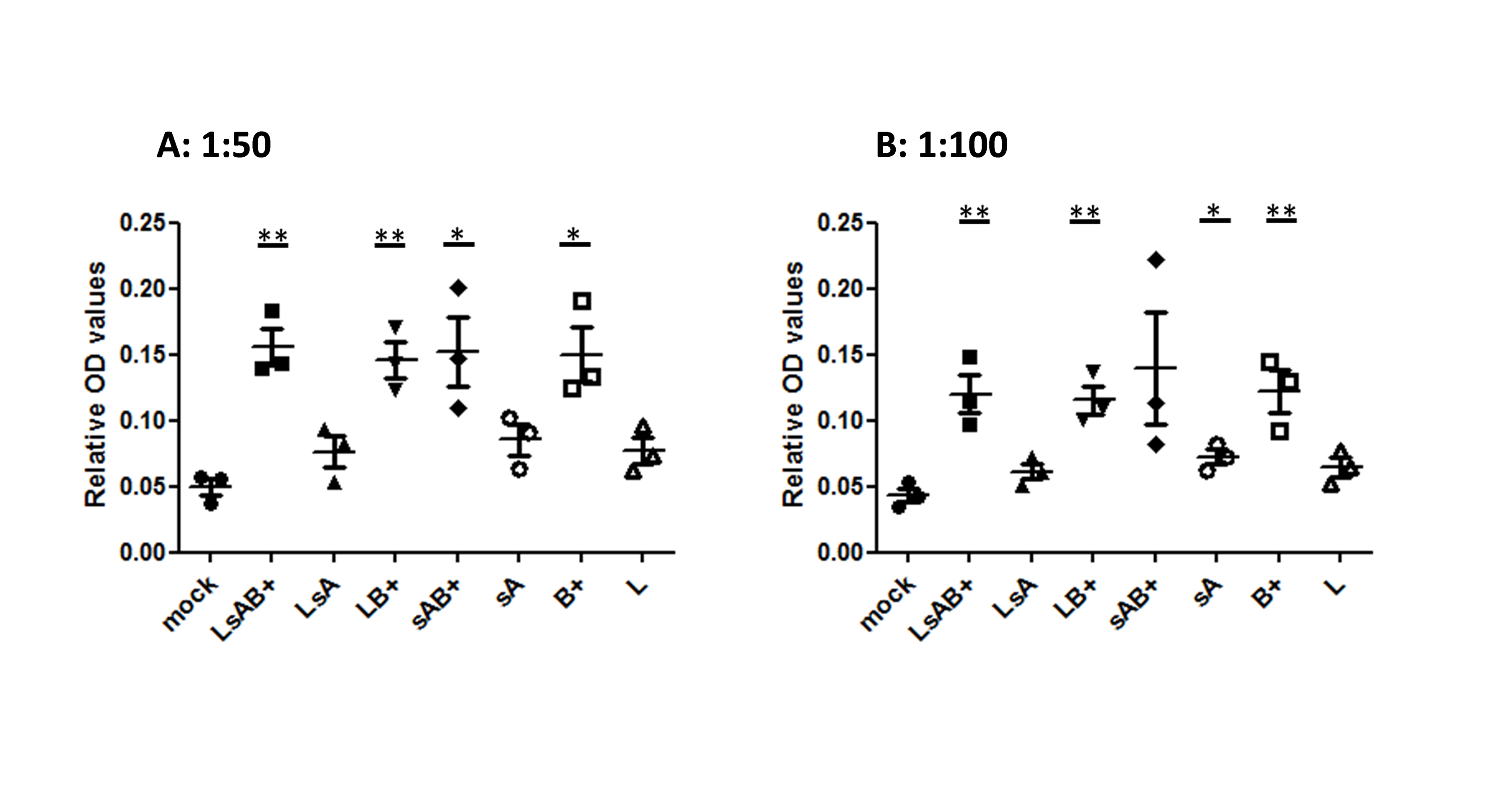

Supplement: S4 Fig — The 96-well microtiter plates were coated with 60 μl CHIKV-AG (Anti-CHIKV virus IgG Elisa kit, Abcam (#ab177835); Cambridge, UK) per well by overnight incubation at 4°C. The plates were washed twice with washing buffer (PBS, 0.05% Tween20) and blocked with blocking buffer (PBS, 1% BSA) for 4 hrs. Afterwards, mouse serum at different dilutions in blocking buffer was added to the wells and the plates were incubated for 1 h at 37°C. Serum of untreated mice was used as negative control. After four times washing, horseradish peroxidase-conjugated goat anti-mouse IgG antibody (Dianova, Hamburg, Germany; 1:5.000) was added and the plates were incubated for 1 h at 37°C. After washing (4 times), 100 μl TMB solution (Tetramethylbenzidine, Interchem, Pfaffen-Schwabenheim, Germany) was used to detect the color development. After incubation for 10 min, the reaction was stopped by adding 100 μl 2N sulfuric acid. The color intensity was measured at 450 nm in a Tecan Genion Plus ELISA Plate Reader and is indicated as relative OD. The immunogens used to vaccinate the mice are indicated on the x-axis. Sera of single mice were used for the ELISA assay and their values are indicated as dots. The bar indicates the mean values of 3 mice vaccinated and the standard deviations are given as vertical bars. P-values calculated by an unpaired-students T-test are indicated as * (P ≤ 0.05) and ** (P ≤ 0.01) and show significance. A: sera were diluted 1:50. B: sera were diluted 1:100. (TIF) [file pntd.0003684.s004.tif]

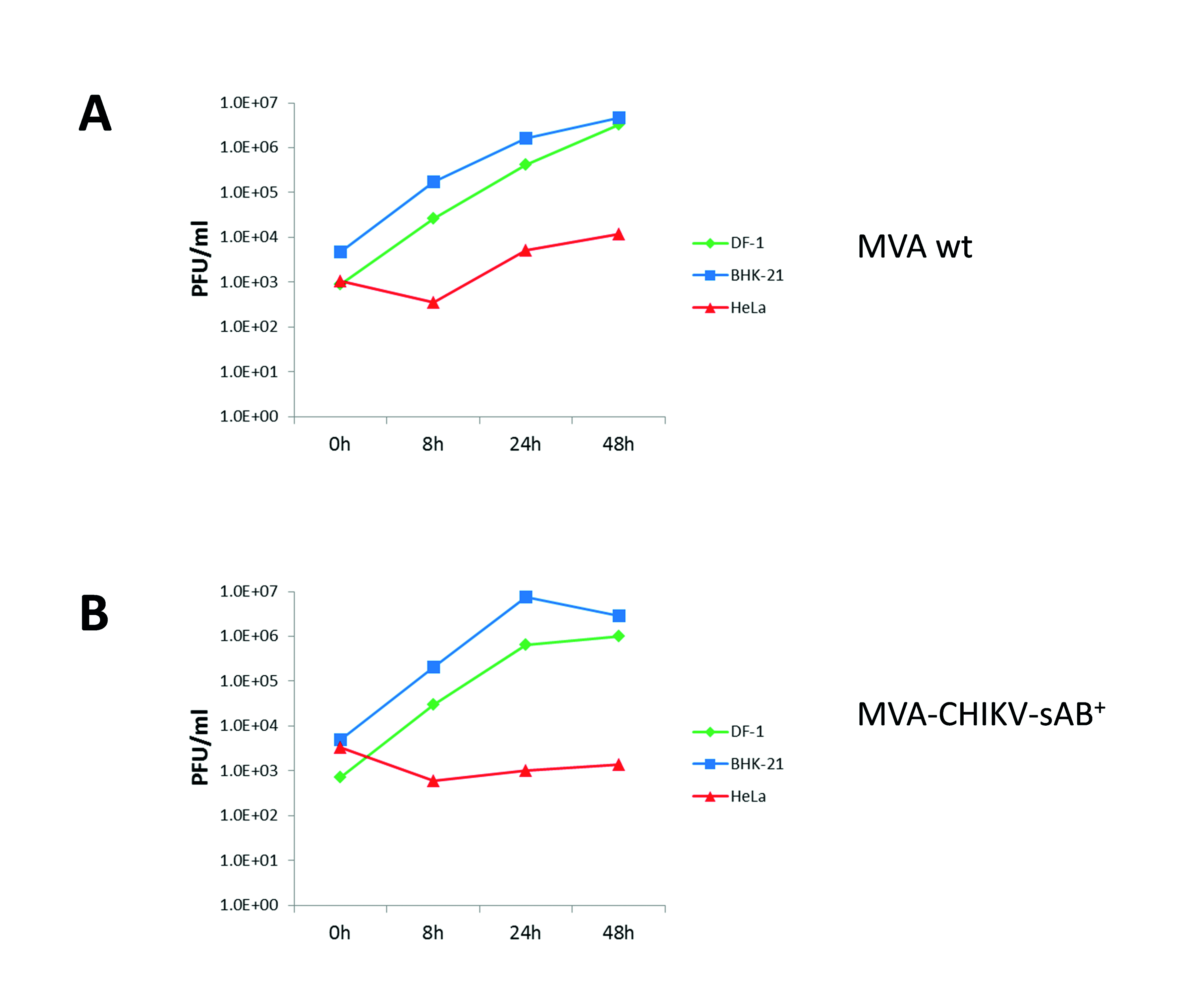

Supplement: S5 Fig — The permissive cell lines DF-1 and BHK-21, and the non-permissive HeLa cells were seeded in 6-well plates and infected with MVA wt (A), or MVA-CHIKV-sAB+ (B) with an MOI of 0.5. Viral titers were determined at the indicated time points. (TIF) [file pntd.0003684.s005.tif]

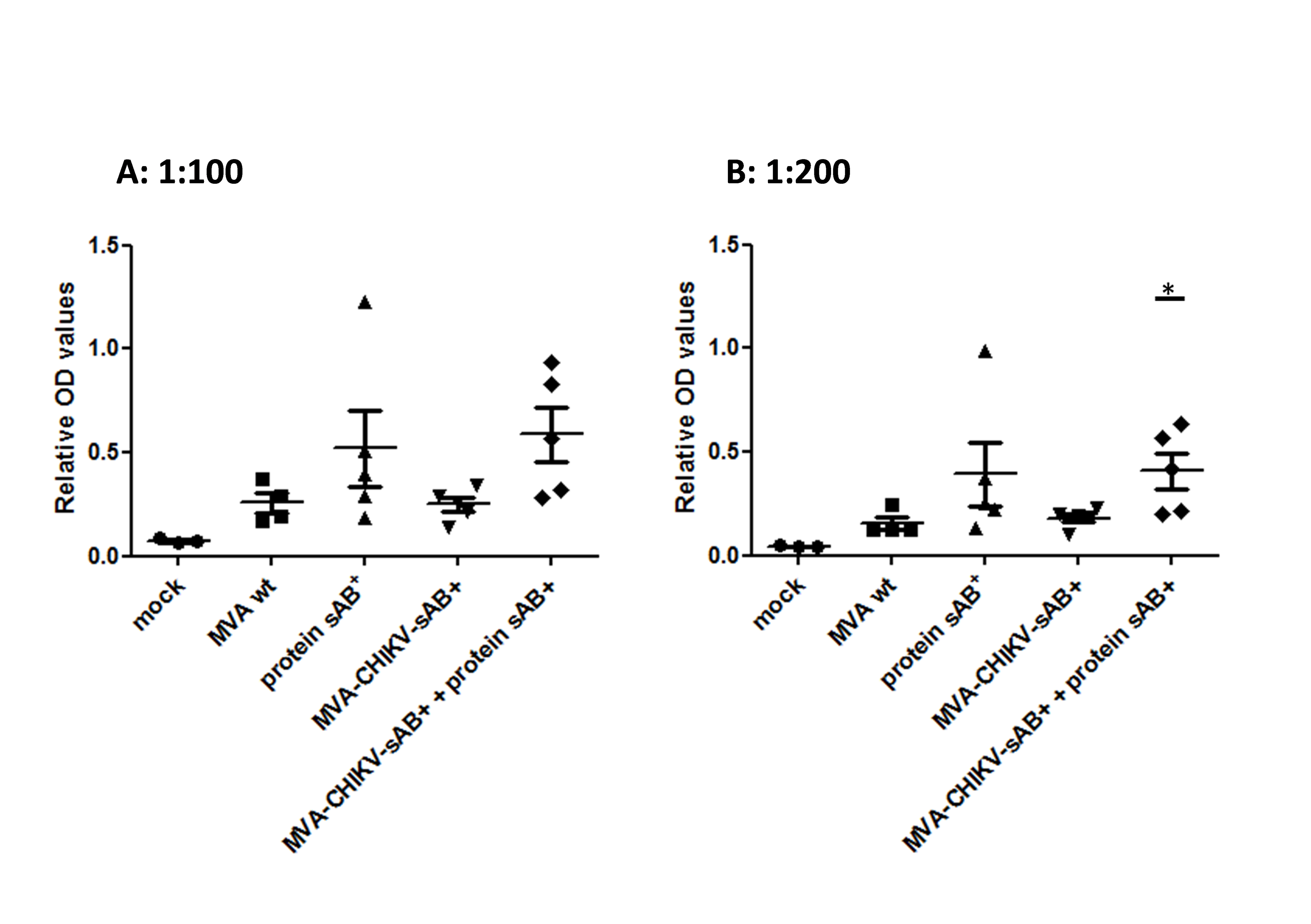

Supplement: S6 Fig — The 96-well microtiter plates were coated with 100 ng protein B+ per well by overnight incubation at 4°C. The plates were washed twice with washing buffer (PBS, 0.05% Tween20) and blocked with blocking buffer (PBS, 1% BSA) for 4 hrs. Afterwards, mouse serum at different dilutions in blocking buffer was added to the wells and the plates were incubated for 1 h at 37°C. Serum of untreated mice was used as negative control. After four times washing, horseradish peroxidase-conjugated goat anti-mouse IgG antibody (Dianova, Hamburg, Germany; 1:5.000) was added and the plates were incubated for 1 h at 37°C. After washing (4 times), 100 μl TMB solution (Tetramethylbenzidine, Interchem, Pfaffen-Schwabenheim, Germany) was used to detect the color development. After incubation for 10 min, the reaction was stopped by adding 100 μl 2N sulfuric acid. The color intensity was measured at 450 nm in a Tecan Genion Plus ELISA Plate Reader and is indicated as relative OD. The immunogens used to vaccinate the mice are indicated on the x-axis. Sera of single mice were used for the ELISA assay and their values are indicated as dots. The bar indicates the mean values of 5 mice (4 for control groups) vaccinated and the standard deviations are given as vertical bars. P-values calculated by an unpaired-students T-test are indicated as * (P ≤ 0.05) and show significance. A: sera were diluted 1:100. B: sera were diluted 1:200. Statistically significance compared to MVA wt vaccinated mice, was only detected for MVA-CHIKV-sAB+/protein sAB+ vaccinated mice. (TIF) [file pntd.0003684.s006.tif]
